# Addressing religious hate online: from taxonomy creation to automated detection

**DOI:** 10.7717/peerj-cs.1128

**Published:** 2022-12-15

**Authors:** Alan Ramponi, Benedetta Testa, Sara Tonelli, Elisabetta Jezek

**Affiliations:** 1 Fondazione Bruno Kessler, Trento, Italy; 2 Dipartimento di Studi Umanistici, Università di Pavia, Pavia, Italy

**Keywords:** Natural language processing, Abusive language detection, Religious hate speech detection

## Abstract

Abusive language in online social media is a pervasive and harmful phenomenon which calls for automatic computational approaches to be successfully contained. Previous studies have introduced corpora and natural language processing approaches for specific kinds of online abuse, mainly focusing on misogyny and racism. A current underexplored area in this context is religious hate, for which efforts in data and methods to date have been rather scattered. This is exacerbated by different annotation schemes that available datasets use, which inevitably lead to poor repurposing of data in wider contexts. Furthermore, religious hate is very much dependent on country-specific factors, including the presence and visibility of religious minorities, societal issues, historical background, and current political decisions. Motivated by the lack of annotated data specifically tailoring religion and the poor interoperability of current datasets, in this article we propose a fine-grained labeling scheme for religious hate speech detection. Such scheme lies on a wider and highly-interoperable taxonomy of abusive language, and covers the three main monotheistic religions: Judaism, Christianity and Islam. Moreover, we introduce a Twitter dataset in two languages—English and Italian—that has been annotated following the proposed annotation scheme. We experiment with several classification algorithms on the annotated dataset, from traditional machine learning classifiers to recent transformer-based language models, assessing the difficulty of two tasks: abusive language detection and religious hate speech detection. Finally, we investigate the cross-lingual transferability of multilingual models on the tasks, shedding light on the viability of repurposing our dataset for religious hate speech detection on low-resource languages. We release the annotated data and publicly distribute the code for our classification experiments at https://github.com/dhfbk/religious-hate-speech.

## Introduction

The growing popularity of social networks has created opportunities for like-minded people to locate each other online and create communities sharing values, as well as social, political or religious views. This is particularly evident on Twitter, where following/follower relations, and reply/quote/retweet interactions ease the creation of more or less connected communities of users (Boyd, Golder & Lotan, 2010; Lee, Hwalbin & Okhyun, 2015; Garimella, Weber & Choudhury, 2016). Despite the positive effects of novel social connections, however, this possibility to bypass traditional media gatekeepers, paired with the high accessibility and potential anonymity of the users, have greatly contributed to the proliferation of online hate (Christopherson, 2007; Johnson et al., 2019). Several types of targets have been identified and analyzed in past literature, from specific individuals such as political candidates (Grimminger & Klinger, 2021) or female videogame makers (Gray, Buyukozturk & Hill, 2017), to groups and categories such as immigrants (Sánchez-Junquera et al., 2021) and journalists (Charitidis et al., 2020).

Given the pervasiveness and magnitude of abusive language online, numerous computational approaches to tackle this problem have been proposed in the past within the NLP community (Fortuna & Nunes, 2018), one of the first being the seminal work in Waseem & Hovy (2016), addressing sexist and racist tweets. Research on online abuse has since then grown to include specific workshops (Waseem et al., 2017a; Kumar et al., 2020; Akiwowo et al., 2020) and shared tasks (Zampieri et al., 2019; Zampieri et al., 2020), and to cover a large number of languages (Carmona et al., 2018; Basile et al., 2019; Corazza et al., 2020; Ranasinghe & Zampieri, 2022). Besides research on online abuse in general terms, some works have tried to categorize different hateful messages online, identifying a number of possible targets and subtargets (Salminen et al., 2018b). Among them, racism and gender-based hatred have been extensively studied, creating also target-specific resources and detection systems. Racism has been analyzed from different perspectives, ranging from detection (Onabola et al., 2021) to annotators’ bias (Sap et al., 2019; Larimore et al., 2021). As regards gender-based hatred online, there have been specific tasks devoted to detecting abusive language towards women (Fersini, Rosso & Anzovino, 2018; Fersini, Nozza & Rosso, 2020), as well as works analyzing gendered stereotypes in automatic sentence completion (Nozza, Bianchi & Hovy, 2021). Misogyny has also been analyzed at a fine-grained level, proposing a novel taxonomy of misogynistic language occurrences (Zeinert, Inie & Derczynski, 2021). Gender issues have been the focus also of the few research activities around automatic detection of microaggressions (Breitfeller et al., 2019). By contrast, religious hate online is a rather understudied problem within the NLP community, despite being an important and impactful societal issue (Albadi, Kurdi & Mishra, 2018). Indeed, according to some studies religious hate can lead to individuals’ radicalization when there is no exposure to information that would challenge these ideas and beliefs (Thompson, 2011; Awan, 2017). It is therefore important to have a better understanding of how religious hate is expressed online, which forms of offensive language are typically employed with this type of target, and whether there are differences across different religions. This would not only lead to a better knowledge of the phenomenon, but also it would allow the development of more accurate and effective systems to detect religious hate online. In this work we therefore focus on this target, considering the world’s three major monotheistic religions: Judaism, Islam and Christianity. To manually and automatically identify religious hate in online conversations, we adapt the definition of Islamophobia provided in Vidgen & Yasseri (2020) to encompass all the targets of interest by defining religious hate as *indiscriminate negative attitudes or emotions directed at a religion or its believers*. Thus, in this study, we consider religious hate as a specific form of abuse or offense against a religious target. Details on our categorization are provided in the “A taxonomy for religious hate” section.

Our main contributions are the following: *(i)* we propose a taxonomy for religious hate covering the three main monotheistic religions, which is aligned with other taxonomies for abusive language classification (Vidgen et al., 2021; Zeinert, Inie & Derczynski, 2021); *(ii)* we create a new dataset containing English and Italian tweets, annotated according to the above taxonomy, which we release for research purposes; and *(iii)* we conduct a set of monolingual and cross-lingual experiments, including zero-shot transfer, to detect abusive language and religious hate speech. Our work shows that religious hate speech detection is a challenging problem even for state-of-the-art pretrained language models (with best results obtained by fine-tuned RoBERTa-based language models (Liu et al., 2019; 65.69 *F*_1_ for Italian, and 64.94 *F*_1_ for English), and highlights the viability of cross-lingual transfer *via* multilingual language models for detecting religious hate speech on languages in which annotated data for the task is not available or easily obtainable (with best results achieved by a fine-tuned XLM-RoBERTa language model (Conneau et al., 2020): 58.53 *F*_1_ for Italian to English, and 60.27 *F*_1_ for English to Italian).

The article is organized as follows. After reviewing related work, we first describe our novel taxonomy to categorize religious hate in online messages, consistent with the taxonomy proposed in Zeinert, Inie & Derczynski (2021) for misogyny and with the categories identified in Vidgen et al. (2021), to which we added “abusive humor”. We then detail the creation of a Twitter dataset containing two subsets to study religious hate, one in English and one in Italian. We describe the data annotation process and provide related documentation in the form of data and artifact statements (Bender & Friedman, 2018; Ramponi & Tonelli, 2022). Finally, we present monolingual and cross-lingual classification experiments on Italian and English data subsets for two tasks: abusive language detection and religious hate speech identification, followed by a quantitative and qualitative analysis of religious hate speech forms across languages and religions, a discussion and our conclusions.

*NOTE: This article contains examples of language which may be offensive to some readers.*

## Related Work

### Taxonomies of online abuse

Since online hate is a complex, multi-faceted phenomenon, the need for a categorization of types of abuse has been addressed in several works, also because it can guide annotators in labeling hate speech data more accurately. Waseem et al. (2017b) present a typology of abusive language, distinguishing between individuals and groups as target and between implicit and explicit hate. A three-layered annotation has been introduced for the OffensEval shared tasks (Zampieri et al., 2020), where participants are required to distinguish between offensive and not offensive tweets, targeted and untargeted offenses, and to classify the target type (*i.e.,* individual, group and other). Other taxonomies include more fine-grained categories of abuse. For example, the annotation scheme proposed by Palmer et al. (2020) revolves around the offensiveness of a message, the presence of slurs, adjectival nominalization, and distancing. Sanguinetti et al. (2018), instead, focus on hate messages against immigrants and annotate hate intensity, aggressiveness, offensiveness, irony and stereotypes. For misogyny, at least two taxonomies have been recently proposed. The first one, by Anzovino, Fersini & Rosso (2018), included five types of misogynistic language, *i.e.,* discredit, stereotype and objectification, sexual harassment, dominance, and derailing. The second one, by Zeinert, Inie & Derczynski (2021), modified the previous one by adding benevolent sexism and neo-sexism and removing derailing, as a consequence of discussions with annotators. Vidgen et al. (2021), instead, propose a general-purpose taxonomy of abusive content, encompassing any type of target and offense type. Abuse, in particular, can be identity-directed, affiliation-directed and person-directed, and the first two categories include derogation, animosity, threatening language, glorification of hateful entities, and dehumanization as sub-categories. Our taxonomy has been designed so to be compliant with Vidgen et al. (2021)’s by further specifying the taxonomy branch related to identity-directed with a religious target. We enrich the subcategories with abusive humor, while merging derogation and dehumanization, and including glorification of hateful entities in other categories depending on context due to their rare occurrence in our dataset.

### Studies on religious hate

The problem of religious hate online and the design of approaches to detect and to counter it are relatively understudied. Among the few works that have focused only on religious targets, Vidgen & Yasseri (2020) present a novel dataset of 4,000 tweets annotated with three classes (*i.e.,* non-Islamophobic, weak Islamophobic and strong Islamophobic) and compare several classification algorithms and feature sets, arguing for a fine-grained classification of hate speech that goes beyond binary classes. Chung et al. (2019) present a large dataset of expert-curated sentence pairs containing Islamophobic messages and counter-narratives. This data has then been used to develop a platform that suggests responses to operators of non-governmental organizations fighting online hatred against Muslims (Chung et al., 2021). Our work is most similar to the study presented in Albadi, Kurdi & Mishra (2018), introducing a dataset of 6,000 annotated tweets in Arabic, where six religious groups (*i.e.,* Jews, Christians, Sunnis, Muslims, Shia and Atheists) are represented with 1,000 tweets each. The work shows that half of the discussions about religion in the Arabic Twittersphere is hateful, targeting in most of the cases Jews, Atheists and Shia. The authors also compare different detection approaches, including lexicon-based, *n*-gram based, and deep learning algorithms. Magdy et al. (2016), instead, deal with stances towards Muslims, collecting tweets about Islam after the Paris terrorist attacks in 2015 and analyzing whether they expressed a positive, negative or neutral stance. As regards antisemitism, Zannettou et al. (2020) quantify the escalation and spread of antisemitic memes and rhetoric across alt-right Web communities. They show that the use of antisemitic terms in Web communities is substantially influenced by real-world events like US Presidential elections. They do not present algorithms for antisemitic message detection but rather focus on lexicon-based analysis through word embeddings. Also, Sabat, Canton-Ferrer & Giró-i-Nieto (2019) investigate hateful memes, considering Jew and Muslim memes, among others. Their detection experiments show that the visual cues are much more important than the linguistic ones when detecting hate speech memes.

Besides the works devoted exclusively to the analysis of religious hate online, a number of datasets include religion as one of the targets (for an overview, see Poletto et al. (2021) and Vidgen & Derczynski (2020)). The corpus described in Vigna et al. (2017) contains Facebook posts in Italian, where religion is one of the categories along with disability, politics, and gender, amongst others. Also, Ishmam & Sharmin (2019) present a Facebook corpus in Bengali where different annotated categories are provided, including religious hatred and religious comments. Along the same line, Mossie & Wang (2020) collect Facebook posts in Amharic covering different topics such as religion, politics, economy and ethnicity. Olteanu et al. (2018), instead, focus on Reddit and Twitter data and analyze how attacks involving Arabs and Muslims trigger online hate speech, and in particular messages advocating violence. Ousidhoum et al. (2019) annotate tweets in English, French and Arabic along speech directness (*i.e.,* direct or indirect), hostility type and target, including religious affiliation.

Overall, existing works related to the classification of religious hate online present some limitations: first of all, Christianity is generally understudied, with the exception of Albadi, Kurdi & Mishra (2018). We find, instead, that Christians are a rather common target, especially in English-written posts. Second, religion has been included in some of the existing taxonomies of hate targets, but no fine-grained categorization has been proposed in the past, distinguishing among different types of targets and hate speech forms, as proposed in our taxonomy. Third, religious hate online has been little explored from a cross-lingual perspective, although the country of origin has an impact on the perceived offensiveness of messages (Salminen et al., 2018a), and religious hate has country-specific cultural and historical roots. Indeed, our classification experiments and the following discussion aim at shedding light also on hate speech forms across religions and languages.

## A taxonomy for religious hate

Since abusive language online is a multi-faceted phenomenon and different categorizations of hate speech have been proposed over the years to account for different targets (Kumar et al., 2018; Vidgen & Yasseri, 2020; Palmer et al., 2020; Zampieri et al., 2020), we aim on the one hand to take the complexity of online abuse into account, and on the other hand to be compatible with existing annotation schemes as much as possible. We therefore build upon the hierarchical taxonomy proposed in Zeinert, Inie & Derczynski (2021), which has been designed to annotate misogynist messages online but nevertheless provides a backbone for the fine-grained annotation of other target types. We chose Zeinert, Inie & Derczynski (2021)’s taxonomy as starting point since it captures a wide range of abuse types and targets, ultimately enabling a high degree of interoperability across annotated datasets. Indeed, although we focus on religious hate speech, we argue that providing annotations even for classes which are not of direct interest in this study (*e.g.*, misogyny, racism) allows data to be easily repurposed in future studies concerning other targets.^1^ 1This is in contrast to the standard practice in datasets with focused targets, in which abusive posts that are not directed to the target under study are typically labeled as not abusive, thus undermining data interoperability for different use cases.

**Figure 1:**
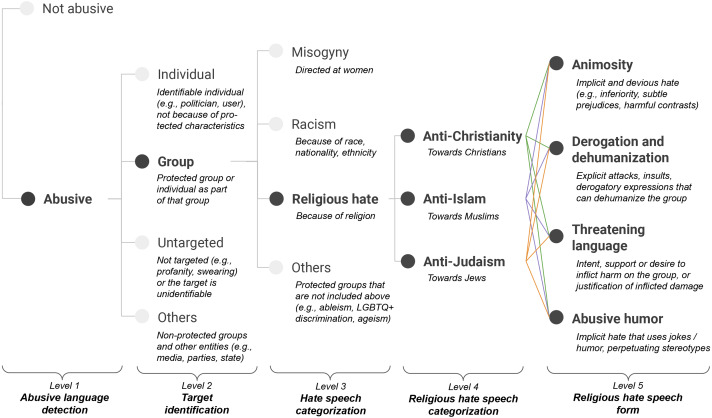
Abusive language annotation taxonomy with a focus on religious hate.

As shown in Fig. 1, we employ the first three levels of Zeinert, Inie & Derczynski (2021)’s taxonomy and extend the last one by adding a religious hate category (cf. “Hate speech categorization” level in Fig. 1), which leads to two fine-grained levels for religious hate speech: (i) a level for fine-grained categorization of religious targets (cf. “Religious hate speech categorization” level in Fig. 1), and (ii) a level for religious hate speech forms (cf. “Religious hate speech form” level in Fig. 1). In the following, we first summarize the first three levels of the taxonomy. Then, we provide definitions and relevant examples for our proposed religious hate levels and associated labels.

### Generic backbone of the taxonomy

In this section, we summarize the first three levels of the taxonomy introduced by Zeinert, Inie & Derczynski (2021), which as mentioned above form the backbone of our annotation scheme for religious hate speech detection.

#### Level 1: Abusive language detection

The top-level of the taxonomy aims at distinguishing whether a post contains abusive language (abusive) or not (not abusive). We rely on the definition by Caselli et al. (2020) for abusive language classification, and consider as abusive any form of hurtful and derogatory language, discriminatory statement targeted at communities, groups or individuals based on personal characteristics, toxic comment, and untargeted profanity. This includes more subtle forms of abusive language, as detailed in the next sections.

#### Level 2: Target identification

Abusive posts can be further classified according to the target to which the abuse is directed. Consistently to Zeinert, Inie & Derczynski (2021), possible labels for this level are group, individual, others and untargeted:

• **group**: the target of the abuse is a *protected* group or an individual as part of that group. We follow the widely-used definition of protected groups (Röttger et al., 2021; Ramponi & Tonelli, 2022; Banko, MacKeen & Ray, 2020), namely groups based on characteristics such as religion, ethnicity, race, gender identity, age, sex or sexual orientation, disability, and national origins. The category is related to Davidson et al. (2017)’s “hate speech” definition and focus on protected characteristics.
• **individual**: the target of the abuse is an identifiable individual and can be either the user to whom one is replying to or a named person that is external to the conversation. The abuse directed to the individual is not because of a protected characteristic.
• **others**: the abuse is targeted at non-protected groups and entities, such as media, institutions, political parties, countries per se, and governments.
• **untargeted**: the abuse is not targeted or the target is unidentifiable. Posts containing profanities, curses, swearing, and otherwise inappropriate language fall into this category.

#### Level 3: Hate speech categorization

Group-targeted abusive posts can be further classified based on the specific protected group the abuse is directed. We take misogyny, racism and others from Zeinert, Inie & Derczynski (2021), and further include a religious hate category.

• **misogyny**: the abuse expressed in the post is directed at women. This includes sexist content, harmful stereotypes, and subtle forms of abuse such as neosexism (Zeinert, Inie & Derczynski, 2021).
• **racism**: the characteristic to which the abuse is targeted is race, nationality or ethnicity of a group. This mainly includes explicit and implicit forms of racism and discrimination.
• **religious hate**: the target of the abuse is a religious group or an individual because of its membership to that community. This includes abuse towards the three main monotheistic religions, namely Christianity, Islam and Judaism (see “Religious hate speech levels” for details).
• **others**: the abuse is directed at protected groups that are not included in the previous categories. These include ableism (discrimination against people with disabilities), LGBTQ+ discrimination (*i.e.,* on the ground of gender identity and sexual orientation), homophobia (discrimination against lesbian, gay, or bisexual people), transphobia (discrimination against transgender people) and ageism (age discrimination).

### Religious hate speech levels

In this section, we present the proposed religious hate speech categories (level 4) and hate forms (level 5). We provide definitions along with relevant examples from our dataset in both English and Italian.

#### Level 4: Religious hate speech categorization

Abusive posts targeted to a religious group (religious hate) can be further characterized based on the specific religion the abuse is directed. As detailed below, we include the main monotheistic religions as religious targets, namely anti-christianity, anti-islam and anti-judaism.

• **anti-christianity**: the target is Christianity and its faithful community. This category includes stereotypes aimed at Christians (*e.g.*, Christians portrayed as murderers or rapists), criticism of subgroups by other Christians, and general contempt for all or part of the Christian community.
  **English example.** “*christians and christianity actively enable sexual abuse environment... the “sexual scandal” involves actual human beings... but all you care about is his legacy and stupid teachings... fuck you very much [URL]*”
  **Italian example.** “*la storia del mio popolo in breve , le parole sono giuste e veritiere, quindi cari cristiani prima di fare le vittime studiate la vostra storia perché avete ancora le mani sporche di sangue [URL]*”—**English translation.** “*the history of my people in short , the words are right and truthful, so dear Christians before playing the victim study your history because your hands are still stained with blood [URL]*”
• **anti-islam**: the abuse is targeted at Islam and its adherents. Common examples include implicit and veiled hatred, dehumanization of Muslims through explicit expressions, using the belonging to Islam as a reason to insult or criticize people’s actions (*e.g.*, those of politicians), the willingness to inflict damage on Muslims or justifying and support already inflicted damages, stereotypes and prejudices (*e.g.*, Muslims as cutthroat), statements about the inferiority of the Islam belief compared to other religions, potentially abusive jokes, and Muslims as invaders.
  **English example.** “*world wake up before you accept muslims in your countries, those people they’re not refugees, they’re all terrorist who want to come to your state and make a plan how to attack, make sure to deport them all back.*”
  **Italian example.** “*l’#islam teme che la scuola gli remi contro. in realtà, l’islam sgozza e decapita i #cristiani in #ue. naturalmente i paladini pro multiculturalismo tutti zitti. [URL]*”—**English translation.** “*#islam fears that the school rows against it. actually, Islam slaughters and beheads #christians in #eu. naturally the pro multicultural champions all shut up. [URL]*”
• **anti-judaism**: the abuse is directed at the Jewish community and its religion. Main examples include the denial of the holocaust and genocide, the explication of religious belief as justification for Jews’ misbehavior, inappropriate comparisons between the Covid-19 pandemic (or vaccines) and the Shoah, insults through explicit swear words or comparisons with inhuman entities (mainly insects), abusive jokes and black humor, stereotypes and prejudices, the desire to inflict damage on the Jews or justification of a violence that has already been inflicted to them by others, and superiority claims at the expense of the Judaism belief.
  **English example.** “*lmaoooo that’s no what happened at all. jews believe that non-jews all a sort of animal created to be their slaves. they’ve treated everyone accordingly for thousands of years which is why so many people have kicked them out [URL]*”
  **Italian example.** “*sicuramente babbo natale è un vecchio paziente e disponibile ma se in regalo gli chiedi di far sparire dalla terra certi ‘parassiti’ (ebrei, of course) il poveretto s’incavola e pure tanto! #antisemitismo*”—**English translation.** “*certainly santa claus is a patient and helpful old man but if you ask him to make certain ‘parasites’ (Jews, of course) disappear from the earth as a gift, the poor man gets pissed off a lot! #antisemitism*”

#### Level 5: Religious hate speech form

Abusive posts that are classified as targeted to a specific religious group on level 4 of the taxonomy are given an additional label indicating the way in which the hatred is manifested. We identify those labels starting from identity-directed abuse labels from Vidgen et al. (2021) (*i.e., derogation*, *animosity*, *threatening language*, *glorification of hateful entities* and *dehumanization*) and conducting a pilot annotation study on Italian data (see “Dataset creation”), which comprises several iterations and group discussions for refining the initial label set. Specifically, first iterations led to religion-specific labels that were iteratively handled and generalized to produce religion-agnostic labels, making the taxonomy more flexible and easier to apply across religious groups. A pivotal example is represented by the *diminishing* label for anti-judaism, initially designed to describe instances of holocaust denial and inappropriate comparisons between the Covid-19 pandemic and the Shoah. We resolved this label by incorporating inappropriate contrasts as cases of animosity (*i.e.,* implicit and soft hatred), whereas we treated holocaust denial differently according to the degree of hatred expressed in the post. We removed *glorification of hateful entities* from our initial label set including those rare cases in the other categories depending on context, and conceived an abusive humor label to encompass instances of offensive jokes and black humor that could cause harm to target religious groups. Our final label set is thus largely based on Vidgen et al. (2021)’s labels and include animosity and threatening language, merges *derogation* and *dehumanization* labels into a single derogation and dehumanization category aiming at reducing ambiguity in annotation, further adding the rather understudied abusive humor category. In the following we define and provide examples for each label of our final set.

• **animosity**: the hatred expressed in the post is implicit and devious, nevertheless it is offensive and humiliating to the target. Implicit hate speech typically employs indirect or coded language (ElSherief et al., 2021), uses rhetorical devices to hide harmful meaning (Caselli et al., 2020), or subtly expresses negativity against certain groups (Vidgen & Yasseri, 2020). This makes identification of animosity highly challenging for automated systems yet a fundamental part of abusive language detection (ElSherief et al., 2021; Caselli et al., 2020). In the context of religious hate speech, this category includes language in which: (a) the religious belief of the abuse target is made explicit, as if to mean that a wrong behavior is due to the religion to which the believer belongs; (b) the superiority of certain religions is manifested at the expense of others (*e.g.*, Christian supremacy); (c) the religious group and its adherents are accused to receive “special and unfair treatments” compared to other groups; (d) a religion is used to criticize the actions of individuals (*e.g.*, politicians, the Pope) in geopolitical areas where another religion prevails; (e) ill-founded criticisms are argued against subgroups of the same religion or adherents to other religions; (f) stereotypes and prejudices about a religious group are perpetuated in a subtle manner without using explicit hate expressions; (g) identity words are used as insult through expressions that introduce presuppositions (*e.g.*, “They’re Muslims/Jews/Christians after all”); (h) inappropriate harmful contrasts between the Covid-19 pandemic and the Shoah are made; (i) holocaust denial is manifested without explicitly insulting or demonizing the target group.
  **English example.** “*[USER] we had the bible and christ before muhammed and the quran , aren’t the latter rather superfluous ?*”
  **Italian example.** “*agli ebrei tatuavano un numero oggi va il qr code. un passo avanti epocale. sono riusciti a farlo fare a loro stessi.*”—**English translation.** “*they tattooed Jews with a number today goes the qr code. an epochal step forward. they managed to get it done to themselves.*”
• **derogation and dehumanization**: the hatred expressed against a target religious group is manifested in an explicit way. The target group is described in negative terms that can also dehumanize it (*e.g.*, adherents presented as animals or otherwise nonhuman entities), together with explicit attacks, insults or offensive and derogatory expressions (*e.g.*, “Islamic cutthroats”). Stereotypes and prejudices that lead to the demonization of the group also fall into this category. This represents one of the most direct and explicit form of hatred.
  **English example.** “*[USER] [USER] #truth add: when muslims kill non-muslims - they stay silent too. hypocrites! wherever islam has infiltrated, it has brought about more harm than good. and we all know it. if it truly is a religion of peace, why has it done the most damage in the world? bunch of sickos! [URL]*”
  **Italian example.** “*[USER] e islamica quindi: popolo sottosviluppato kattivo invidioso stupratore spacciatore con l’esclusiva del terrorismo.*”—**English translation.** “*she’s Islamic so: underdeveloped bad people envious rapist drug dealer with the exclusive right of terrorism.*”
• **threatening language**: this category includes explicit forms of hateful language in which the willingness to inflict harm on a religious group is manifested, an already inflicted damage is justified or supported, or someone is encouraged to inflict such damage. In line with Vidgen et al. (2021), harm includes physical and emotional violence, harassment and social exclusion. threatening language represents the most explicit and dangerous case of online hatred.
  **English example.** “*[USER] [USER] from just muslim to ahmadi muslim, something new, something innovative, something never seen or heard before, nothing like the ahmadis had happened before, that means you made a new religion, let alone a sect, thats why you are apostates who deserve d3ath*”
  **Italian example.** “*[USER] per combatterli bisogna mandarli a casa loro tutti i musulmani dico tutti buoni e cattivi non ne ne importa nulla cos risolviamo sicuramente il@problema e poi si decapitassero tra di loro*”—**English translation.** “*[USER] to fight them we must send them back to their home all the Muslims all good and bad it doesn’t matter so we definitely solve the@problem and let them behead each other*”
• **abusive humor**: an understudied case of implicit hate which uses jokes and black humor, indirectly leading to the stereotyping of target religious groups, and potentially perpetuating prejudices and causing harm to such groups. We treat it as a new and separate category since offensive jokes against religious groups are common in online fora, and the characteristics of such language are different from other cases of implicit language, being often sarcastic and metaphorical.
  **English example.** “*i think my life is like a 12 yo boy in a catholic church cus man it do be sucking...*”
  **Italian example.** “*[USER] la differenza tra un ebreo e una torta? il tempo di cottura*”—**English translation.** “*[USER] the difference between a Jew and a cake? cooking time*”

## Dataset Creation

In this section, we present the protocol we followed for collecting and annotating religious hate speech data in English and Italian. We then provide documentation in the form of data and artifacts statements (Bender & Friedman, 2018; Ramponi & Tonelli, 2022), as well as summary statistics and insights about the annotated corpus. While data collection follows the same protocol for both languages, we adopt two different approaches to data annotation.

For Italian, we had access to three native speakers with a background in computational linguistics, therefore annotation was performed following a protocol for experts that foresaw in-person discussion rounds and adjudication sessions.^2^ 2This was also motivated by the scarcity of Italian workers on crowdsourcing platforms such as Amazon Mechanical Turk.Furthermore, the assignment of multiple labels was allowed to account for intersectionality when more than one target type was found in a tweet. For English, we could not follow the same approach, because we did not have the possibility to recruit English native speakers. However, we believe that being a native speaker should be a mandatory requirement for annotators to fully capture the subtleties and the cultural references in our domain of interest. We therefore resort to Amazon Mechanical Turk, following a standard workflow to collect multiple judgments from crowd-workers, while taking advantage of the EasyTurk tool (Bocchi, Frasnelli & Aprosio, 2021), that enables a strict quality control and monitoring of annotators’ performance. In this case, however, the annotation protocol was slightly simplified, removing the possibility to assign multiple labels.

Although following exactly the same annotation process for the two languages would have been ideal to allow cross-lingual comparisons, mixing annotation approaches has already been investigated in prior work and has proven to have a limited impact on annotation quality (Waseem, 2016; Sanguinetti et al., 2018). In particular, Waseem (2016) found that cases of high-agreement provided by amateur annotators lead to relatively good annotations as compared to expert annotators, making the crowd-sourcing protocol a viable approach in the case of complex multi-stage annotation schemes or absence of native speakers.

### Data collection

We collected tweets for both English and Italian using the Twitter APIs by querying for key terms which are likely to occur in religious posts and refer to the three main monotheistic religions, namely Christianity, Islam and Judaism (cf. Appendix). The list of search keywords has been designed with and validated by domain experts involved in religious studies, as there were neither accessible nor complete lexicons to rely on at the time of collection. In order to make English and Italian portions of the dataset comparable, native or proficient English and Italian speakers curated term translations between the two languages.

We specifically avoid to use offensive words as part of our search terms since they are known to bias the dataset towards explicit rather than implicit abusive language (Wiegand, Ruppenhofer & Kleinbauer, 2019), and instead rely only on neutral terms with reference to specific religions, their branches, adherents, and sacred texts (*e.g.*, “Islam”, “Catholicism”, “Jewish”, “Sunni”, “Torah”). Although this has the downside of producing a lower proportion of abusive language in the resulting dataset (Founta et al., 2018), religion on online fora is a highly debated topic, and thus naturally leads to a high proportion of abusive language. Furthermore, to reduce as much as possible the diachronic bias of our dataset (Florio et al., 2020), we collected data spanning a nine-month-long time period (December 2020 – August 2021), thus mitigating the over-representation of online discourse about religion-related historical events.

Data collection has been performed in September 2021. After collection, we randomly sampled 3,000 Italian posts and 8,000 English posts for further abusive language annotation.

### Data annotation

We devise annotation guidelines as described in “Taxonomy of religious hate” to ensure consistency in the labeling process across annotators and languages. Each post has been presented to annotators in an anonymized form and classified out-of-context with up to five labels (*i.e.,* until a leaf node is reached) corresponding to the levels of the taxonomy depicted in Fig. 1.

#### Italian subset.

As mentioned above, the annotation for the Italian portion of the dataset has been performed by three expert annotators. All annotators are native speakers of Italian and have computational linguistics and computer science backgrounds. Two annotators identify themselves as females and one as male, with age ranges 20–30, 30–40 and 40–50. All annotators have a Catholic background, albeit being Atheists or non-practicing Christians. At the first stage of annotation, all annotators have been involved in discussion sessions to revise and validate the proposed labeling scheme and associated annotation guidelines. In order to enable intersectionality studies in the future, expert annotators provided multiple labels per instance during labeling, if applicable. To assess the quality of annotations, 20% of the dataset has been annotated by two annotators. We computed inter-annotator agreement using Cohen’s kappa (Cohen, 1960) for the two categorizations of interest to this work, namely *abusiveness* (*i.e.,* abusive *vs* not abusive), and *religion-related abusiveness* (*i.e.,* all posts with a religious hate label in level 3 of the taxonomy *vs* all others). We obtain *κ* = 0.6530 for abusiveness, and *κ* = 0.5651 for religion-related abusiveness before adjudication, which is moderate agreement. This is in line with other abusive language datasets (Zeinert, Inie & Derczynski, 2021) and reflects the difficulty of annotation for the task, which in our case was mainly due to a different interpretation of the tone of a given tweet. Then, cases of disagreement between the two annotators have been discussed and adjudicated by the third annotator. The resulting Italian portion of the dataset consists of 3,000 labeled examples.

#### English subset.

The English portion of the dataset has been annotated by native English speakers from the United States, United Kingdom and Australia, following the annotation guidelines previously refined and validated for the Italian subset, using Amazon Mechanical Turk. Each tweet has been annotated by up to five annotators. To ensure high-quality annotations from non-expert crowd-workers and thus preventing potential scams, we follow Leonardelli et al. (2021), and ask three expert linguists to annotate a batch of examples, then using tweets with perfect agreement as gold standard. We include a gold tweet in each group of ten examples to be annotated, using EasyTurk (Bocchi, Frasnelli & Aprosio, 2021). If a crowd-worker fails to evaluate the gold standard, we discard the remaining worker’s annotations from our dataset. About 3% of the workers’ annotations were removed from the study. Annotators were compensated on average with 8 US$ per hour. In order to make the annotation for the full taxonomy as clear and manageable as possible for the non-expert crowd, we proceed as follows. First, we ask crowd-workers to annotate the first two levels of the taxonomy (level 1 and 2 in Fig. 1). This allows us to identify potentially abusive messages towards religion (*i.e.,* those having majority group label in level 2) to be further annotated. Second, annotators provide labeling decisions on group-labeled tweets for the third and fourth level of the taxonomy (level 3 and 4 in Fig. 1). Although our quality control largely ensures spam annotations are not retained in the dataset, we argue that the subjective nature of the annotation task could lead to disagreement on a small fraction of previous decisions, and thus we give annotators the possibility to express their disagreement on previous annotations. We then remove from our final dataset the instances marked by the majority of annotators as disagreement. Third, we ask annotators to label the instances exhibiting anti-christianity, anti-islam or anti-judaism majority labels for the last level of the taxonomy. We include in the final dataset all examples having at least three individual annotations and a label majority on all non-leaf annotation stages. Furthermore, to minimize noisy annotations from the non-expert crowd, annotators have been instructed to only mark the main category at each annotation stage. As a result, the final English portion of the dataset consists of 7,028 fully labeled tweets.

We computed the inter-annotator agreement and use Krippendorff’s alpha (Krippendorff, 2004) to account for multiple raters and sparse annotations. This led to *α* = 0.2667 for *abusiveness*, which is in line with agreement reported on previous datasets employing complex annotation schemes (Ousidhoum et al., 2019; Sanguinetti et al., 2018), and *α* = 0.8386 for *religion-related abusiveness*, which is high agreement due to the multi-stage annotation workflow we devise for the English portion of the dataset. Since we adopt the strict quality-control protocol implemented through the EasyTurk application (Bocchi, Frasnelli & Aprosio, 2021), it is unlikely that disagreement is due to spammers and low-quality annotations, but it is rather due to different annotators’ attitudes and background (Aroyo & Welty, 2015). Recent works advocate for reporting annotators’ information when releasing datasets with toxicity annotation to account for biases and disagreement cases (Sap et al., 2022), but the processing of personal data concerning religious beliefs is explicitly prohibited by the EU General Data Protection Regulation (GDPR) (https://eur-lex.europa.eu/legal-content/EN/TXT/HTML/?uri=CELEX:32016R0679&from=EN#d1e40-1-1), therefore we did not collect any such information from crowd-workers. For the sake of transparency, we release disaggregated labels for the full dataset, so to enable future research on annotators’ disagreement.

### Dataset documentation

In this section, we present relevant documentation for our dataset based on data and artifacts statements (Bender & Friedman, 2018; Ramponi & Tonelli, 2022).

#### Data statements

Data statements are provided in order to have a better overview of the rationale of creating the dataset and the taxonomy. According to Bender & Friedman (2018), data statements represent a professional practice capable of “*provid[ing] context to allow developers and users to better understand how experimental results might generalize, how software might be appropriately deployed, and what biases might be reflected in systems built on the software*”. There are not only scientific implications involved, but ethical issues as well. Indeed, data statements for NLP alleviate issues related to exclusion and bias in language technology. In the following, we present data statements for the dataset we introduce in this work.

##### Curation rationale.

The dataset was collected using neutral keywords related to religion (see Appendix) specifically avoiding offensive search terms that are known to bias the dataset towards explicit hate messages (Wiegand, Ruppenhofer & Kleinbauer, 2019). The dataset comprises an Italian and an English subset, and was created to the purpose of studying and mitigating the spread of online religious hate. A data instance is a tweet annotated according to a religious hate taxonomy scheme (see “Taxonomy for religious hate”).

##### Language varieties.

The variety of Italian (it-IT) and English (en-*, *i.e.,* without distinction between regional variants) represented in the dataset is spontaneous written speech.^3^ 3Many labels have been proposed to account for the characteristics of web varieties (Guardiano, 2007). All labels (*talky writing*, *conversational writing*, *electronic discourse*, etc.) agree with considering web varieties a hybrid form of communication with characteristics of both written and spoken language, to which such exclusive features as redundant punctuation, abbreviations, etc. are added.

##### Speaker demographic.

Data consists of anonymized posts, and thus user demographics are unknown.

##### Annotator demographic.

*Italian subset.* Three native speakers of Italian, two females and a male, with age ranges 20–30, 30–40, and 40–50. All annotators have computational linguistics and computer science education, and a Catholic background (albeit being Atheists or non-practicing Christians). *English subset.* The pool of annotators are English speakers from the United States, United Kingdom and Australia. Other demographic characteristics have not been disclosed and are thus unknown.

##### Speech situation and text characteristics.

The interaction is mainly asynchronous, the speaker’s intended audience is everyone, and the text genre is social media with a focus on religious topics. Posts have been produced between December 2020 and August 2021 and collected in September 2021.

##### Preprocessing and data formatting.

Posts have been anonymized by replacing user mentions and URLs with [USER] and [URL] placeholders, respectively. Texts have been preprocessed by lowercasing the text, removing newline characters, and unescaping possible HTML tags.

#### Artifacts statement

Lexical artifacts statement has been introduced by Ramponi & Tonelli (2022) as a way to document potential lexical biases when a dataset is released, providing a complementary view to data statements (Bender & Friedman, 2018). Lexical artifacts are defined as emergent correlations between tokens and labels in input data. We outline the statement for our dataset as follows.

##### Top lexical artifacts.

In Table 1, we present the top *k* = 15 lexical artifacts for the abusive and religious hate classes for Italian and English data subsets.

**Table 1:** Top 15 most informative tokens for the abusive class (left) and religious hate class (right) on Italian and English data subsets. Note that in some cases the lists capture some of the prejudices and stereotypes related to religion, see for example ‘invasione’ (invasion, mainly with respect to Muslims), ‘finanza’ (finance, mainly with respect to Jews), rape, terrorists, and ‘9/11’. Emojis credit: OpenMoji—the open-source emoji and icon project (CC BY-SA 4.0).

Rank	Abusive		Religious hate	
	Italian subset	English subset	Italian subset	English subset
1	Cazzo	Fuck	Islamica	Kill
2	Bergoglio	Fucking	Mica	Fuck
3	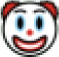 (*“Clown”*)	Kill	Islam	Cult
4	Fascisti	Fake	Invasione	Raped
5	Islamica	Ass	Portare	Jews
6	Invasione	Shit	Mussulmani	Jew
7	Mica	Rape	Maomettani	Representation
8	Merda	Stupid	Cazzo	Rape
9	Schifo	Hating	Nuovi	Terrorists
10	Sionisti	Representation	Islamici	Fake
11	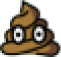 (*“Poo”*)	Disgusting	Rabbino	Strange
12	Papa	Terrorist	Finanza	9/11
13	Islamici	Assholes	Clandestini	Cum
14	Dittatura	Biggest	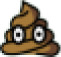 (*“Poo”*)	Hypocrites
15	Vaccinati	Pedophiles	Islamico	Ugly

##### Class definitions.

The definition for abusive and religious hate classes are presented in “A taxonomy for religious hate” section.

##### Methods and resources.

The *k* = 15 most informative tokens for the classes of interest have been extracted from the top features of a bag-of-words logistic regression model, in line with Kennedy et al. (2020). Stopwords have been removed using the Stopwords ISO resource (https://github.com/stopwords-iso). Prior to computation, input texts have been preprocessed as described in “Preprocessing and data formatting”. Tokens have been produced using the spaCy v3.3 tokenizer (https://spacy.io/) employing it_core_news_sm and en_core_web_sm models for Italian and English, respectively.

### Dataset statistics

The final dataset consists of 10,028 annotated tweets, of which 3,000 are in Italian and 7,028 in English. The average tokens per post are 33.5 for Italian and 34.9 for English. Dataset statistics across taxonomy levels and languages are reported in Table 2. For Italian, we report between parenthesis the total count of annotations, given that it was possible to assign multiple labels to the same tweet. For English, numbers within parenthesis in level 5 indicate the number of annotations considering multiple majority labels. The other count, instead, includes only tweets annotated with exactly one label.

**Table 2:** Dataset statistics across taxonomy levels and languages. Numbers outside parentheses indicate tweets having exactly that label, whereas numbers between parentheses are total counts per label.

	Class	Total	Italian	English
*Level 1*	abusive	1,961	954	1,007
	not abusive	8,067	2,046	6,021
*Level 2*	group	1,130 (1,312)	404 (586)	726
	individual	315 (412)	154 (251)	161
	untargeted	115 (129)	48 (62)	67
	others	193 (323)	140 (270)	53
	*multiple labels*		208	–
*Level 3*	religious hate	1,163 (1,220)	500 (557)	663
	misogyny	10 (24)	7 (21)	3
	racism	42 (86)	14 (58)	28
	others	40 (51)	8 (19)	32
	*Multiple labels*		57	–
*Level 4*	anti-christianity	304 (313)	85 (94)	219
	anti-islam	591 (603)	326 (338)	265
	anti-judaism	310 (322)	131 (143)	179
	*Multiple labels*		15	–
*Level 5*	animosity	720 (781)	369 (381)	351 (400)
	derogation and dehumanization	139 (390)	139 (141)	196 (249)
	threatening language	35 (46)	17 (18)	18 (28)
	abusive humor	51 (67)	17 (17)	34 (50)
	*Multiple labels*		15	–

Since the tweets were collected using the same set of keywords in the two languages, it is possible to some extent to compare the content of the two subsets. Overall, we observe that the proportion of abusive messages in Italian (31.8%) is much higher than in English (14.3%). Also the distribution of offenses against the three religions of interest is different: while in English they are more balanced (33% target Christianity, 40% Islam and 27% Judaism), in Italian most of the offenses are Islamophobic (16% are against Christians, 60% Muslims and 24% Jews). This would confirm the findings in Ljujic et al. (2015), reporting that Italy is the most Islamophobic country in Europe after Hungary, and also the analysis provided by the Italian Observatory on Human Rights (http://www.voxdiritti.it/la-nuova-mappa-dellintolleranza-6/), showing that in 2021 the two groups most targeted on Twitter were women and Muslims. Concerning level 5, in both languages the cases of animosity are the most frequent, *i.e.,* mild or not overt abuse. Threats instead are the least frequent, also because they are prohibited by law in many countries, including Italy and UK.

In the Italian subset, annotators have often exploited the possibility to assign multiple labels to a tweet. We identify two main reasons for multiple assignments: (i) the presence of more than one target in the same message, for instance a politician and a religious group (see example 1 below), and (ii) a group targeted because of multiple sensitive attributes, for example Muslim women (see example 2 below).^4^ 4Examples have been slightly edited.The first cases are by far the most frequent.

(1) “*La Boldrini fedelissima dei talebani! Gli islamici la votano perché agevola il lavoro di islamizzazione alla velocità della luce!*”—**English translation.** “*Boldrini, the most loyal to the talibans! Islamics vote for her because she facilitates the work of Islamization at the speed of light!*”

(2) “Le donne islamiche dicono che scegliendo tale religione allora scelgono anche di mettersi il velo e di avere relazioni solo con musulmani ma allora scegliete anche di andarvene aff**”—**English translation.** “Islamic women say that by choosing this religion they also choose to wear the veil and have relations only with Muslims, but then you also choose to go f*ck yourself”

## Experiments

In this section, we present the experimental setup and the classification methods we used for conducting experiments on the annotated dataset in both languages. We perform two binary classification tasks: one is aimed at abusive language detection (level 1 in Table 2), namely identifying posts that contain abusive language, and the second at religious hate speech detection (level 3 in Table 2), *i.e.,* a fine-grained classification task aiming at detecting posts expressing religious hate.

### Experimental setup

We cast both detection tasks as binary classification problems, in which the two classes to be predicted are abusive and not abusive for abusive language detection, whereas are religious hate and not religious hate for religious hate speech detection. For the sake of experiments, in religious hate speech detection we consider all tweets that do not exhibit a religious hate label for the not religious hate class. This leads to a total of 557 (18.57%) and 663 (8.26%) religious hate examples for the Italian and English subsets, respectively. For abusive language detection, the number of abusive posts are 954 (31.80%) for Italian and 1007 (14.33%) for English. We preprocess all tweets by anonymizing user mentions and URLs with [USER] and [URL] placeholders, respectively. We then lowercase the text, remove newline characters, and unescape HTML tags.

Given the unbalanced distribution of labels across tasks and languages, we use macro-averaged precision (Prec), recall (Rec) and F1 score (*F*_1_) as main metrics to assess the performance of our classifiers. This allows us to reliably evaluate performance of classification methods by giving the minority class equal importance to the majority one, and thus mitigating performance overestimation of other commonly used metrics (*e.g.*, accuracy (Acc), that we also present for the sake of completeness). For all our experiments, we use stratified *k*-fold cross-validation (*k* = 5) and report mean and standard deviation of all scores.

We perform experiments under two classification setups: a monolingual setup, in which all classifiers are trained and tested on in-language data, and a cross-lingual setup, in which classifiers are either (a) trained on both languages and tested on a target language (*i.e.*, cross-lingual learning), or (b) trained and tested on different languages (*i.e.*, zero-shot cross-lingual transfer). For the challenging yet more interesting cross-lingual setup, we employ multilingual pretrained language models, as described in the next section.

### Classification algorithms

In order to gauge the level of difficulty of our tasks, we perform experiments using several classification methods, from naïve baselines and traditional machine learning classifiers to language-specific and multilingual pretrained language models.

#### Naïve baselines.

We test three simple baselines to assess the complexity of both binary tasks: (i) always abusive (or always religious hate), which always predicts a tweet as abusive (or religious hate), (ii) always not abusive (or always not religious hate), which always predicts a tweet as not abusive (or not religious hate), and (iii) random, which simply predicts a label at random. Although being trivial, those baselines serve as comparison for more complex classifiers, and thus enable to determine if more elaborate solutions are able to capture useful features to tackle both tasks.

#### Machine learning classifiers.

We employ four traditional machine learning classifiers as implemented in the scikit-learn library (https://scikit-learn.org/stable/). Those algorithms are (i) decision tree, (ii) multinomial naïve Bayes, (iii) linear support vector classifier, and (iv) logistic regression. We use the scikit-learn count vectorizer for feature extraction and employ default hyperparameters as detailed in the official documentation across all our experiments. These baselines serve for comparison purposes to more recent solutions based on fine-tuning of monolingual or multilingual pretrained language models.

#### Language-specific pretrained language models.

We experiment with transformer-based (Vaswani et al., 2017) language models pretrained on monolingual raw data, and fine-tune them on both binary tasks. For English, we employ (i) BERT (Devlin et al., 2019), whose pretraining corpus consists of 16GB of English text data (roughly 3.3B words) from BooksCorpus (Zhu et al., 2015) and English Wikipedia, and (ii) RoBERTa (Liu et al., 2019), whose pretraining procedure included 160GB of English raw text (*i.e.,* with additional news and web texts to BERT’s pretraining corpus). We use the bert-base-uncased and roberta-base model versions, respectively, due to the relatively small size of our annotated datasets. We refer the reader to the original publications for details on different pretraining schemes of the two approaches (Devlin et al., 2019; Liu et al., 2019). For Italian, we use (i) AlBERTo (Polignano et al., 2019), a BERT-based language model pretrained on 191GB of text from a collection of tweets in Italian, and (ii) UmBERTo (umberto-commoncrawl-cased-v1 version) (https://huggingface.co/Musixmatch/umberto-commoncrawl-cased-v1), a RoBERTa-based language model pretrained on the deduplicated Italian portion of the OSCAR corpus (Ortiz Suárez, Sagot & Romary, 2019),^5^ 5OSCAR is a large-scale multilingual corpus based on filtered Common Crawl data that has been classified by language.which accounts for a total of 70GB of raw text (rougly 11B words). We use all models as implemented in the MaChAmp v0.2 toolkit (van der Goot et al., 2021b) using default hyperparameters (Table 3), and a cross-entropy loss with balanced class weights to give equal importance to both classes.

**Table 3:** Hyperparameter values used in our experiments for fine-tuning pretrained language models.

Hyperparameter	Value
Optimizer	AdamW
*β*_1_, *β*_2_	0.9, 0.99
Dropout	0.3
Epochs	3
Batch size	32
Learning rate (LR)	5e−5
LR scheduler	Slanted triangular
Decay factor	0.38
Cut fraction	0.2

#### Multilingual pretrained language models.

For the sake of cross-lingual experiments, we employ two multilingual pretrained language models that include both Italian and English in the pretraining corpus: (i) multilingual BERT (Devlin et al., 2019), a BERT model pretrained on the 104 languages with the largest Wikipedia, and (ii) XLM-RoBERTa (Conneau et al., 2020), a RoBERTa-based model pretrained on 2.5TB of CommonCrawl raw text containing 100 languages. We use the bert-base-multilingual-cased and xlm-roberta-base versions, respectively, as implemented in the MaChAmp v0.2 toolkit (van der Goot et al., 2021b) with default hyperparameters (Table 3). As for monolingual pretrained language models, we use cross-entropy loss with balanced class weights.

## Results and Analysis

In this section, we present monolingual and cross-lingual results and analysis of different classifiers across binary tasks and languages.

### Monolingual classification setup

We here present the results under the monolingual setup, namely when training and testing classifiers on the same language (either the Italian or English subset).

#### Italian.

In Tables 4 and 5 we report the *k*-fold cross validation results for abusive language detection and religious hate speech detection, respectively, on the Italian subset. Traditional machine learning classifiers generally improve the performance over naïve baselines, with logistic regression and linear support vector classifier being the most effective approaches of the category (67.21 and 66.23 *F*_1_ score for abusive language detection, and 59.76 and 60.89 *F*_1_ score for religious hate speech detection, respectively). Noticeably, multinomial naïve Bayes provides high precision scores, at the cost of a fairly low recall across tasks. However, best results for both tasks are obtained by fine-tuned language models, and specifically by monolingual models, with results ranging from 75.02 to 76.31 *F*_1_ score for abusive language detection and from 64.86 to 65.69 *F*_1_ score for religious hate speech detection. Interestingly, multilingual language models still outperform traditional machine learning classifiers according to the *F*_1_ score metric. Indeed, multilingual BERT achieves 73.46 and 61.49 *F*_1_ score, whereas XLM-RoBERTa reaches 71.37 and 63.90 *F*_1_ score. Overall, the best results for both abusive language detection and religious hate speech detection are obtained by the fine-tuned UmBERTo model, which outperforms all other language models across all metrics. Although AlBERTo uses pretraining data pertaining to a domain that is closer to our dataset than UmBERTo (*i.e.,* Twitter texts), the consistent improvement of UmBERTo over AlBERTo suggests that the RoBERTa’s pretraining scheme employed by UmBERTo is more beneficial on our tasks than the domain and size of the raw data used for pretraining.

**Table 4:** *k*-fold cross validation results for abusive language detection on the Italian subset. Results are averages (with standard deviation as subscript). Precision, recall, and *F*_1_ scores are macro-averages. Best results for metrics of interest are in bold.

Method	Acc	Prec	Rec	*F*_1_
Always abusive baseline	31.80 ± 0.1	15.90 ± 0.1	50.00 ± 0.0	24.13 ± 0.1
Always not abusive baseline	68.20 ± 0.1	34.10 ± 0.1	50.00 ± 0.0	40.54 ± 0.1
Random baseline	47.80 ± 2.9	48.11 ± 2.1	47.83 ± 2.5	46.17 ± 2.6
Decision tree	67.17 ± 1.1	61.10 ± 1.4	59.98 ± 1.1	60.31 ± 1.2
Multinomial naïve Bayes	74.47 ± 0.5	72.28 ± 0.9	64.19 ± 1.0	65.17 ± 1.2
Linear support vector classifier	72.10 ± 1.6	67.36 ± 2.1	65.64 ± 2.1	66.23 ± 2.2
Logistic regression	73.97 ± 1.4	70.00 ± 1.9	66.23 ± 1.7	67.21 ± 1.8
AlBERTo	77.20 ± 2.2	74.35 ± 2.1	76.57 ± 2.1	75.02 ± 2.2
UmBERTo	78.53 ± 1.1	**75.68**± 1.0	**77.72**± 1.4	**76.31**± 1.1
Multilingual BERT	76.50 ± 2.4	73.17 ± 2.5	74.08 ± 3.0	73.46 ± 2.7
XLM-RoBERTa	72.83 ± 1.4	72.02 ± 1.3	74.86 ± 2.0	71.37 ± 0.9

**Table 5:** *k*-fold cross validation results for religious hate speech detection on the Italian subset. Results are averages (with standard deviation as subscript). Precision, recall, and *F*_1_ scores are macro-averages. Best results for metrics of interest are in bold.

Method	Acc	Prec	Rec	*F*_1_
Always religious hate baseline	18.57 ± 0.1	9.28 ± 0.1	50.00 ± 0.0	15.66 ± 0.1
Always not religious hate baseline	81.43 ± 0.1	40.72 ± 0.1	50.00 ± 0.0	44.88 ± 0.1
Random baseline	48.83 ± 3.1	49.38 ± 1.9	48.98 ± 3.2	43.56 ± 2.7
Decision tree	77.60 ± 1.0	60.68 ± 2.4	58.88 ± 2.4	59.50 ± 2.4
Multinomial naïve Bayes	81.73 ± 0.4	**73.62**± 7.9	51.57 ± 0.7	48.32 ± 1.2
Linear support vector classifier	79.53 ± 1.1	63.72 ± 2.1	59.78 ± 1.3	60.89 ± 1.5
Logistic regression	81.73 ± 0.6	68.17 ± 2.5	58.36 ± 1.3	59.76 ± 1.6
AlBERTo	72.77 ± 2.8	64.34 ± 1.1	71.08 ± 1.9	64.86 ± 1.8
UmBERTo	73.57 ± 2.1	64.87 ± 1.8	**71.86**± 2.6	**65.69**± 2.2
Multilingual BERT	68.83 ± 9.5	61.98 ± 5.5	67.77 ± 7.9	61.49 ± 8.2
XLM-RoBERTa	73.87 ± 1.9	63.08 ± 1.4	67.75 ± 3.1	63.90 ± 1.7

#### English.

In Tables 6 and 7 we report the *k*-fold cross validation results for abusive language detection and religious hate speech detection on the English portion of the dataset. Similarly to monolingual experiments on the Italian subset, language-specific pretrained language models provide the highest overall scores on both tasks. The best results according to macro-averaged precision, recall and *F*_1_ score are achieved by RoBERTa, which consistently outperforms BERT achieving 72.56 *F*_1_ score on abusive language detection and 64.94 *F*_1_ score on religious hate speech detection. Results on the English subset, whose distribution is even more skewed than the Italian one towards the negative classes (*i.e.,* not abusive and not religious hate), also reveal the weakness of the accuracy metric for assessing the performance on our tasks. For instance, an “always not abusive” (or “always not religious hate”) baseline seems to provide higher performance than most of the methods, albeit being unable to predict abusive (or religious hate) examples. Overall, we notice lower performance on the English subset compared to the Italian subset despite the former being larger. We hypothesize this could be due to a more varied language use across users that write in English on Twitter, the different annotation methodology, or the more challenging distribution of classes in the dataset.

**Table 6:** *k*-fold cross validation results for abusive language detection on the English subset. Results are averages (with standard deviation as subscript). Precision, recall, and *F*_1_ scores are macro-averages. Best results for metrics of interest are in bold.

Method	Acc	Prec	Rec	*F*_1_
Always abusive baseline	14.33 ± 0.1	7.16 ± 0.1	50.00 ± 0.0	12.53 ± 0.1
Always not abusive baseline	85.67 ± 0.1	42.84 ± 0.1	50.00 ± 0.0	46.14 ± 0.1
Random baseline	49.64 ± 1.3	50.21 ± 1.2	50.43 ± 2.4	42.67 ± 1.4
Decision tree	81.94 ± 0.4	59.61 ± 1.0	57.13 ± 0.9	57.93 ± 1.0
Multinomial naïve Bayes	85.91 ± 0.2	77.49 ± 6.2	51.30 ± 0.8	48.85 ± 1.5
Linear support vector classifier	83.32 ± 1.0	64.04 ± 2.4	61.11 ± 1.8	62.23 ± 2.1
Logistic regression	85.29 ± 0.6	67.42 ± 2.8	58.17 ± 1.3	59.98 ± 1.0
BERT	83.25 ± 1.5	69.55 ± 1.5	77.45 ± 1.9	72.01 ± 1.6
RoBERTa	83.25 ± 1.7	**69.96**± 1.6	**78.77**± 1.2	**72.56**± 1.6
Multilingual BERT	78.40 ± 3.2	65.91 ± 1.6	75.57 ± 2.0	67.55 ± 2.3
XLM-RoBERTa	80.08 ± 1.4	67.32 ± 1.1	77.66 ± 1.0	69.58 ± 1.4

**Table 7:** *k*-fold cross validation results for religious hate speech detection on the English subset. Results are averages (with standard deviation as subscript). Precision, recall, and *F*_1_ scores are macro-averages. Best results for metrics of interest are in bold.

Method	Acc	Prec	Rec	*F*_1_
Always religious hate baseline	9.43 ± 0.1	4.72 ± 0.1	50.00 ± 0.0	8.62 ± 0.1
Always not religious hate baseline	90.57 ± 0.1	45.28 ± 0.1	50.00 ± 0.0	47.52 ± 0.1
Random baseline	49.16 ± 1.6	49.81 ± 1.0	49.43 ± 2.9	39.61 ± 1.4
Decision tree	86.64 ± 0.5	54.29 ± 1.0	52.90 ± 0.9	53.21 ± 1.0
Multinomial naïve Bayes	90.52 ± 0.1	55.29 ± 20.	50.04 ± 0.2	47.66 ± 0.3
Linear support vector classifier	87.07 ± 0.2	57.55 ± 1.3	55.56 ± 1.5	56.23 ± 1.5
Logistic regression	89.68 ± 0.5	61.03 ± 4.6	53.02 ± 1.7	53.46 ± 2.5
BERT	81.87 ± 1.7	62.70 ± 0.8	74.52 ± 1.3	64.93 ± 1.0
RoBERTa	81.46 ± 1.4	**62.71**± 1.0	**75.38**± 1.9	**64.94**± 1.3
Multilingual BERT	75.95 ± 2.5	59.68 ± 0.6	72.40 ± 0.7	60.05 ± 1.5
XLM-RoBERTa	78.53 ± 4.0	60.20 ± 1.2	71.19 ± 3.9	61.15 ± 2.1

### Cross-lingual classification setup

We here present the results under the cross-lingual setup, namely cross-lingual learning (*i.e.,* training multilingual models on both languages, then testing them on a target language), and zero-shot cross-lingual transfer (*i.e.,* training and testing multilingual models on different languages).

#### Cross-lingual learning.

We investigate the ability of multilingual pretrained language models (namely, multilingual BERT and XLM-RoBERTa) on abusive language detection and religious hate speech detection for a target language – either Italian (*IT*) or English (*EN*) – when trained on both language subsets (*i.e., IT+EN*). This allows us to empirically determine if more data, even if it belongs to different languages, provides useful signals to models in the training process. Consistently to monolingual experiments, target language data is partitioned into *k* = 5 80%/20% train/test splits, and each model is thus evaluated *k* times on the *k* test portions. As shown in Table 8 (top), for abusive language detection in English (*IT+EN* → *EN*) both fine-tuned language models improve the *F*_1_ performance over the respective variants trained on English language only (cf. Table 6). The improvement is as large as 2.79 *F*_1_ score for multilingual BERT and 1.52 *F*_1_ score for XLM-RoBERTa. When testing those models on the Italian subset (*IT+EN* → *IT*), however, we notice only a small improvement for XLM-RoBERTa (0.44 *F*_1_ score), whereas a performance degradation occurs for multilingual BERT (−1.83 *F*_1_ score) (cf. Table 4). Regarding religious hate speech detection (Table 8 (bottom)), we notice a similar trend in the performance of models. Specifically, results on English test data (*IT+EN* →*EN*) show an improvement of 2.65 *F*_1_ score for multilingual BERT and 1.40 *F*_1_ score for XLM-RoBERTa (cf. Table 7), whereas results on Italian test data (*IT+EN* → *IT*) confirm that multilingual models struggle in generalizing across languages on the task (cf. Table 5). Multilingual BERT slightly improves the performance when training on additional English data (0.60 *F*_1_ score), whereas XLM-RoBERTa exhibits substantially lower scores (−3.19 *F*_1_ score). Overall, monolingual models provide better performance across tasks and setups (cf. Tables 4, 5, 6 and 7), suggesting that multilingual models can be primarily leveraged in *zero-shot cross-lingual* setups, as described in the next section.

**Table 8:** *k*-fold cross validation results for cross-lingual abusive language detection and religious hate speech detection. Results are averages (with standard deviation as subscript). Precision, recall, and *F*_1_ scores are macro-averages. Best results for metrics of interest and setups are in bold.

Setup	Method	Acc	Prec	Rec	*F*_1_
***Abusive language detection***					
*IT*+*EN*→*EN*	Multilingual BERT	82.03 ± 1.2	68.01 ± 1.3	75.83 ± 1.5	70.34 ± 1.4
	XLM-RoBERTa	82.95 ± 1.8	**68.99**± 1.3	**75.95**± 2.4	**71.10**± 1.1
*IT*+*EN*→*IT*	Multilingual BERT	72.90 ± 3.2	72.00 ± 2.2	75.15 ± 2.4	71.63 ± 2.9
	XLM-RoBERTa	72.93 ± 1.5	**72.38**± 1.7	**75.74**± 2.0	**71.81**± 1.6
***Religious hate speech detection***					
*IT*+*EN*→*EN*	Multilingual BERT	80.49 ± 1.7	60.97 ± 0.9	**71.53**± 1.3	**62.70**± 1.3
	XLM-RoBERTa	82.60 ± 4.1	**61.83**± 1.1	69.36 ± 7.1	62.55 ± 2.1
*IT*+*EN*→*IT*	Multilingual BERT	68.03 ± 1.6	**63.30**± 0.6	**71.36**± 1.0	**62.09**± 1.1
	XLM-RoBERTa	66.60 ± 3.3	62.41 ± 1.8	69.93 ± 2.9	60.71 ± 2.7

#### Zero-shot cross-lingual transfer.

In real-world setups, there is typically a lack of annotated data for a particular task on a target language. Since our dataset contains two language subsets, we simulate a *zero-shot cross-lingual* setup, assuming we only have data from a given *source language* and thus aiming at classifying data on a *target language*. In Table 9 we report the results of our experiments across tasks (*i.e., abusive language detection* and *religious hate speech detection*) and languages (*i.e., IT* → *EN* and *EN* → *IT*). Overall, we notice that XLM-RoBERTa provides the best overall performance across our languages and tasks. By a closer look, zero-shot *abusive language detection* results on English data (*IT* →*EN*) reach 63.56 *F*_1_ score, whereas zero-shot results on Italian data (*EN* → *IT*) achieve 70.57 *F*_1_ score (Table 9 (top)). This is remarkable, especially for XLM-RoBERTa on the *EN* → *IT* setup, whose performance are near to the XLM-RoBERTa variant trained on in-language data (−0.80 *F*_1_ score) and not too far from results of the in-language UmBERTo language model (−5.74 *F*_1_ score) (cf. Table 4). As regards *religious hate speech detection*, XLM-RoBERTa results under the *EN* → *IT* zero-shot setup compared to results when using in-language data exhibits a 3.63 *F*_1_ score degradation (cf. Table 5), whereas for the *IT* → *EN* setup the performance drop is even smaller (*i.e.,* −2.62 *F*_1_ score) (cf. Table 7). Noticeably, the difference between XLM-RoBERTa on a zero-shot setup compared to the best monolingual model is −6.41 *F*_1_ score for the *IT* →*EN* setup, and −5.42 *F*_1_ score for the *EN* → *IT* setup. This suggests that zero-shot cross-lingual learning is a viable option for both abusive language detection and religious hate speech detection when annotated data on a target language is unavailable. This finding is in contrast with previous works showing that zero-shot settings do not yield satisfying results for cross-lingual hate speech detection (Nozza, 2021). Our experiments suggest that adopting exactly the same sampling procedures in terms of query terms and time period makes the subsets of tweets in the two languages comparable, and this similarity is probably captured well by multilingual transformer models.

**Table 9:** *k*-fold cross validation results for zero-shot cross-lingual abusive language detection and religious hate speech detection. Results are averages (with standard deviation as subscript). Precision, recall, and *F*_1_ scores are macro-averages. Best results for metrics of interest and setups are in bold.

Setup	Method	Acc	Prec	Rec	*F*_1_
***Abusive language detection***					
*IT*→*EN*	Multilingual BERT	85.05 ± 0.4	**66.49**± 2.3	58.97 ± 3.9	60.33 ± 4.8
	XLM-RoBERTa	78.27 ± 5.2	64.66 ± 2.0	**69.24**± 7.3	**63.56**± 3.1
*EN*→*IT*	Multilingual BERT	73.23 ± 1.7	69.42 ± 2.5	66.96 ± 2.5	67.41 ± 2.1
	XLM-RoBERTa	75.40 ± 1.4	**71.75**± 1.8	**69.96**± 1.2	**70.57**± 1.2
***Religious hate speech detection***					
*IT*→*EN*	Multilingual BERT	82.71 ± 3.9	56.61 ± 2.7	58.09 ± 3.3	56.79 ± 3.0
	XLM-RoBERTa	84.35 ± 3.6	**58.87**± 1.4	**60.54**± 4.8	**58.53**± 3.1
*EN*→*IT*	Multilingual BERT	73.63 ± 2.4	59.68 ± 2.0	61.50 ± 3.8	59.96 ± 2.5
	XLM-RoBERTa	72.20 ± 3.3	**60.20**± 1.0	**63.11**± 3.6	**60.27**± 0.6

## Discussion

In this section we provide a quantitative and qualitative analysis of annotated tweets in the last two levels of the taxonomy (Fig. 1), specifically investigating religious hate forms across religions and languages. Finally, we present a discussion on limitations of our study and directions for future work.

### Analysis of religious hate speech forms across religions and languages

In Table 10 we present the proportion of religious hate speech forms across languages and religions in our dataset. By analyzing data qualitatively, we observe some differences in the distribution of hate forms depending on the targeted religion. Islam appears to be the religion with the greatest number of derogation and dehumanization tweets across languages (30.8% for Italian and 39.4% for English). It is generally portrayed as hostile, dangerous and threatening for the West, or mentioned within the narration of immigration and integration. Muslim hatred is especially evident in the Italian subset (Table 2). We hypothesize that the main reason for this different distribution is that the Italian portion of the dataset contains tweets that are mainly produced by a single nation, where the majority of citizens declares to be Catholic (https://www.uaar.it/doxa2019/), whereas the English subset comprises tweets produced by speakers from all over the world. For this reason, we argue that it is also possible to find more systematic hateful patterns in the Italian tweets. For instance, ‘islamico’ (Islamic) has started being used as a noun with a negative connotation. Moreover, only in the Italian data subset the reference to Islam is often exploited to criticize politicians and the Pope. Overall, the distribution of hate labels in our dataset reflects the anti-Muslim sentiment that is on the rise across Western countries, and whose growth has been analyzed by social scientists in recent work (Cervi, Tejedor & Gracia, 2021).

**Table 10:** Distribution of religious hate speech forms across languages and religions.

	**Italian**			**English**		
	Christianity	Islam	Judaism	Christianity	Islam	Judaism
animosity	71.4%	63.1%	77.9%	63.2%	56.2%	56.4%
derogation and dehumanization	25.3%	30.8%	12.1%	29.2%	39.4%	27.6%
threatening language	2.2%	3.8%	4.3%	1.9%	2.8%	4.3%
abusive humor	1.1%	2.3%	5.7%	5.7%	1.6%	11.7%

Hatred towards Jews, on the contrary, appears to be more implicit and subtle across languages. Specifically, Judaism is the religion that exhibits the largest proportion of abusive humor tweets in both Italian and English. A highly common pattern in the Italian data is the occurrence of inappropriate juxtapositions between Covid-19 themes (*e.g.*, vaccines) and the Shoah, which we treat as animosity in our annotation guidelines. Prejudices, either overtly malevolent (*i.e.,* derogation and dehumanization) or ostensibly benign (*i.e.,* animosity) (see Wilson (1996) for more details), are also relatively frequent in both languages.

Finally, derogation and dehumanization tweets are more frequent in anti-christianity tweets than anti-judaism ones, and slightly less frequent than anti-islam posts. Moreover, according to a qualitative exploration of our labeled dataset, reference to Christianity is often used to affirm religion supremacy at the expense of other religions. Overall, we notice that the distribution of religious hate speech targeting Christianity is similar across languages, with Italian tweets expressing more animosity and English tweets containing a larger fraction of abusive humor.

### Limitations and future work

We acknowledge some limitations of this study. First, our dataset focuses on the three main monotheistic religions of the world, adopting a Western-centric approach. In future work we aim to extend our dataset to additional religions following our religious hate speech annotation scheme, and further include other languages from diverse families. Second, our annotations are provided out-of-context, however a fraction of the posts could be more reliably labeled if the thread context is taken into account in the annotation process. We aim to investigate the impact of contextual information in future work, along the line of Vidgen et al. (2021) and Menini, Aprosio & Tonelli (2021). Third, we acknowledge that our dataset could embed subtle biases due to both the data sampling procedure and annotators’ background. For data collection, even if we designed search keywords with experts in religious studies, deliberately avoiding to rely on offensive word lists, our sampling procedure is still purposive (Wiegand, Ruppenhofer & Kleinbauer, 2019). Since a more desirable random sampling is often impractical due to the low presence of abusive posts on Twitter (Founta et al., 2018), in future work we aim to investigate alternative, half-way data sampling techniques such as keyword expansion after seed terms bootstrapping to further diversify our dataset. As regards annotation, the Italian portion of the dataset has been labeled by expert annotators with Christianity background due to difficulties in finding expert annotators who are both native speakers of Italian and whose belief is Judaism or Islam. However, annotators’ lived experiences, sensibility and faith play a role in how they could perceive religious hate. In order to ultimately enable to take into account as many annotators’ perspectives as possible in religious hate speech detection, we decided to release disaggregated annotations in our dataset. We believe this could enable research directions in modeling different annotators’ perspectives, following successful applications in subjective tasks (Davani, Díaz & Prabhakaran, 2022; Ramponi & Leonardelli, 2022), as well as smoothly provide valuable extensions to our dataset.

Interesting future avenues for research include also studying the impact of lexical normalization on downstream abusive language detection and religious hate speech detection performance, using monolingual (van der Goot et al., 2020; Baldwin et al., 2015) or multilingual datasets (van der Goot et al., 2021a), as well as exploiting multiple annotations on the Italian portion of the dataset to study intersectionality.

## Conclusions

In this work we present an in-depth analysis of religious hate as expressed in Twitter data in Italian and English. We first introduce a taxonomy with five different annotation layers, which has been designed to be fully compatible with the taxonomy for misogyny annotation presented in Zeinert, Inie & Derczynski (2021). The taxonomy covers the three main monotheistic religions as well as different types of offense, from the more subtle ones (animosity) to the very explicit threatening language. Based on this categorization, we collect English and Italian tweets using neutral religion-related terms, and annotate them using two different approaches: for English, we rely on a standard workflow collecting crowd-worker judgments through Amazon Mechanical Turk, whereas for Italian the tweets are annotated by three native speakers going through discussion and adjudication sessions when needed. Experts are also given the possibility to assign multiple labels to the same tweets, opening future avenues for research on intersectionality. Using the resulting dataset, we perform experiments by comparing different classification algorithms in two binary tasks: the detection of abusive language, and the identification of religious hate. Our results show that monolingual pretrained language models provide the best performance in both tasks, and that zero-shot cross-lingual transfer is a viable option for religious hate speech detection when annotated data for the target language is not available.

## Appendix

**Keywords**

**English**: Islam, muslim, muslims, moslem, moslems, islamic, islamics, quran, qur’an, koran, sunnah, hadith, athar, sunni, sunnism, shia, shi’a, shiism, shi’ism, sunnis, sunnite, sunnites, shias, shi’as, shiite, shi’ite, shiites, shi’ites, judaism, jew, jews, jewish, jewishes, jewry, jewries, tanach, hebrew bible, torah, midrash, talmud, rabbanite, rabbinic, rabbinism, rabbinicism, karaite, qaraite, karaism, qaraism, rabbanites, rabbi, rabbis, rabbinist, rabbinists, rabbinicist, rabbinicists, karaites, qaraites, christianity, christians, bible, old testament, new testament, catholicity, catholicism, catholic church, protestantism, eastern orthodox church, orthodox catholic church, catholic, catholics, protestant, protestants, orthodox, orthodoxes.

**Italian**: Islam, musulmano, musulmana, mussulmano, mussulmana, musulmani, musulmane, mussulmani, mussulmane, maomettano, maomettana, maomettani, maomettane, islamico, islamica, islamici, islamiche, corano, quran, qur’an, koran, sunna, sunnah, hadith, athar, sunnismo, sciismo, sunnita, sunniti, sciita, sciiti, ebraismo, giudaismo, ebreo, ebrea, ebrei, ebree, ebraico, ebraica, ebraici, ebraiche, tanakh, tenakh, bibbia ebraica, torah, torà, midrash, talmud, rabbinico, rabbinismo, caraita, karaita, karaismo, karaitismo, caraismo, caraitismo, rabbino, rabbina, rabbini, rabbine, rabbinista, rabbinisti, rabbiniste, caraiti, karaiti, cristianesimo, cristiani, bibbia, antico testamento, vecchio testamento, primo testamento, antica alleanza, nuovo testamento, nuova alleanza, cattolicità, cattolicesimo, chiesa cattolica, protestantesimo, chiesa ortodossa orientale, chiesa ortodossa d’oriente, chiesa cristiana orientale, chiesa cristiana d’oriente, chiesa cattolica ortodossa, cattolico, cattolica, cattolici, cattoliche, protestante, protestanti, ortodosso, ortodossa, ortodossi, ortodosse.

## Additional Information and Declarations

### Data Availability

The following information was supplied regarding data availability: The data and code are available on Zenodo: Ramponi, Alan, Testa, Benedetta, Tonelli, Sara, & Jezek, Elisabetta. (2022). Resources for the article: “Addressing religious hate online: From taxonomy creation to automated detection” (v1.0). Zenodo. https://doi.org/10.5281/zenodo.7082068.
